# NF-*κ*B/AP-1-Targeted Inhibition of Macrophage-Mediated Inflammatory Responses by Depigmenting Compound AP736 Derived from Natural 1,3-Diphenylpropane Skeleton

**DOI:** 10.1155/2014/354843

**Published:** 2014-10-19

**Authors:** Van Thai Ha, Heung Soo Beak, Eunji Kim, Kwang-Soo Baek, Muhammad Jahangir Hossen, Woo Seok Yang, Yong Kim, Jun Ho Kim, Sungjae Yang, Jeong-Hwan Kim, Yung Hyup Joo, Chang Seok Lee, Joonho Choi, Hong-Ju Shin, Sungyoul Hong, Song Seok Shin, Jae Youl Cho

**Affiliations:** ^1^Department of Genetic Engineering, Sungkyunkwan University, Suwon 440-746, Republic of Korea; ^2^Medical Beauty Research Institute, AmorePacific R&D Center, Yongin 446-729, Republic of Korea; ^3^Department of Animal Science, Patuakhali Science and Technology University, Patuakhali 8602, Bangladesh

## Abstract

AP736 was identified as an antimelanogenic drug that can be used for the prevention of melasma, freckles, and dark spots in skin by acting as a suppressor of melanin synthesis and tyrosinase expression. Since macrophage-mediated inflammatory responses are critical for skin health, here we investigated the potential anti-inflammatory activity of AP736. The effects of AP736 on various inflammatory events such as nitric oxide (NO)/prostaglandin (PG) E_2_ production, inflammatory gene expression, phagocytic uptake, and morphological changes were examined in RAW264.7 cells. AP736 was found to strongly inhibit the production of both NO and PGE_2_ in lipopolysaccharide- (LPS-) treated RAW264.7 cells. In addition, AP736 strongly inhibited both LPS-induced morphological changes and FITC-dextran-induced phagocytic uptake. Furthermore, AP736 also downregulated the expression of multiple inflammatory genes, such as inducible NO synthase (iNOS), cyclooxygenase- (COX-) 2, and interleukin- (IL-) 1*β* in LPS-treated RAW264.7 cells. Transcription factor analysis, including upstream signalling events, revealed that both NF-*κ*B and AP-1 were targeted by AP736 via inhibition of the IKK/I*κ*B*α* and IRAK1/TAK1 pathways. Therefore, our results strongly suggest that AP736 is a potential anti-inflammatory drug due to its suppression of NF-*κ*B-IKK/I*κ*B*α* and AP-1-IRAK1/TAK1 signalling, which may make AP736 useful for the treatment of macrophage-mediated skin inflammation.

## 1. Introduction

The skin harbors a complex immunological defence system composed of various immune and nonimmune cells such as epithelial cells, macrophages, keratinocytes, mast cells, and Langerhans cells [1]. Of all these cell types, the most powerful immune cells that reside in the skin are macrophages [1, 2]. These cells comprise an important arm of defence against infections with various Gram (+) or Gram (−) bacteria, fungi, or viruses; macrophages are also important in the defence against other environmental stresses such as chemicals, radiation, pollutants, and ultraviolet (UV) light, all of which can cause skin inflammation [3–6]. These immunogens and irritants are known to activate macrophages, which in turn release various inflammatory mediators and cytokines, including nitric oxide (NO), tumour necrosis factor- (TNF-) *α*, interleukin- (IL-) 1, cyclooxygenase- (COX-) 2, and matrix metalloproteinases (MMPs) [7–10]. Upon encountering these cytokines, neighbouring macrophages activate complicated intracellular signalling cascades involving the activation of nonreceptor protein tyrosine kinases and serine-threonine protein kinases, such as mitogen-activated protein kinases (e.g., p38, extracellular signal-regulated kinase [ERK], and c-Jun N-terminal kinase [JNK]), as well as the stimulation of inflammation-linked transcription factors such as nuclear factor- (NF-) *κ*B and activator protein- (AP-) 1 [11–13].

**Figure 1:**
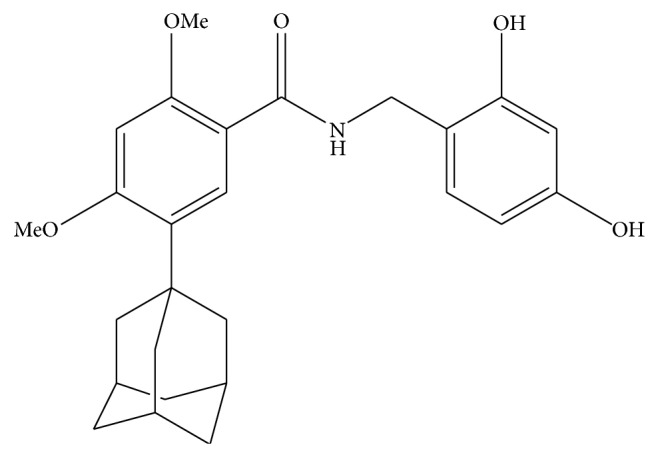
Chemical structure of AP736.

The development of new products for successfully controlling melanogenesis has long been a goal of skin researchers [14]. Although melanin plays a critical role in protecting the skin from UV irradiation, an excess release of melanin from melanocytes can cause melasma, freckles, and dark spots [15]. Therefore, antimelanogenesis remedies would potentially be very valuable to the cosmetic and pharmaceutical industries. In this context, the small molecule AP736 (Figure 1) was recently synthesized from a 1,3-diphenylpropane skeleton through an optimization process involving the replacement of a phenyl group by dihydroxyl and adamantyl moieties [16]; importantly, AP736 was found to effectively suppress melanin production from melanocytes [17]. Although AP736 is clearly an effective depigmenting drug [16], it has not yet been determined whether it can suppress macrophage-mediated skin inflammatory responses, which are crucial for skin health [1]. The aim of this study was thus to determine the precise effect of AP736 on the proinflammatory roles of macrophages stimulated with G (−) bacterium-derived lipopolysaccharide (LPS) and to carefully elucidate the anti-inflammatory mechanisms of AP736.

## 2. Materials and Methods

### 2.1. Materials

AP736 [5-adamantan-1-yl-N-(2,4-dihydroxybenzyl)-2,4-dimethoxybenzamide, 97.5% purity] was synthesized according to previously described methods [16]. Polyethylenimine (PEI), 3-(4,5-dimethylthiazol-2-yl)-2,5-diphenyltetrazolium bromide (MTT), sodium nitroprusside (SNP), 2′7′-dichlorodihydrofluorescein diacetate (DCF-DA), fluorescein isothiocyanate- (FITC-) dextran, indomethacin, *N*_*ω*_-nitro-L-arginine methyl ester hydrochloride (L-NAME), and lipopolysaccharide (LPS;* E. coli* 0111:B4) were purchased from Sigma Chemical Co. (St. Louis, MO, USA). BAY11-7082, U0126 (U0), and SP600125 (SP) were obtained from Calbiochem (La Jolla, CA, USA). The enzyme immune assay (EIA) kits that were used to determine PGE_2_ levels were purchased from Amersham (Little Chalfont, Buckinghamshire, UK). Fetal bovine serum and RPMI1640 were obtained from Gibco (Grand Island, NY, USA). The murine macrophage cell line RAW264.7 and the human embryonic kidney cell line HEK293 were purchased from ATCC (Rockville, MD, USA). All other chemicals were of analytical grade and were obtained from Sigma. Luciferase constructs containing binding sites for NF-*κ*B and AP-1 were gifts from Professor Hae Young Chung (Pusan National University, Pusan, Korea). Phosphospecific and total antibodies to NF-*κ*B subunits (p65 and p50), AP-1 family proteins (c-Fos and c-Jun), I*κ*B*α*, AKT, ERK, p38, JNK, mitogen-activated protein kinase (MKK)4/7, MEK1/2, TAK1, ITAK-1, IRAK-4, lamin A/C, and *β*-actin were obtained from Cell Signaling (Beverly, MA, USA).

### 2.2. Cell Culture

RAW264.7 and HEK293 cells were cultured in RPMI 1640 medium supplemented with 10% heat-inactivated fetal bovine serum (FBS; Gibco, Grand Island, NY, USA), glutamine, and antibiotics (penicillin and streptomycin) at 37°C under 5% CO_2_. Cells were detached with a cell scraper for all experiments. When cells were cultured for experiments at 2 × 10^6^ cells/mL, the proportion of dead cells was less than 1% as determined by Trypan blue dye exclusion.

### 2.3. Determination of NO and PGE_2_ Production

After preincubation of RAW264.7 cells or peritoneal macrophages (1 × 10^6^ cells/mL) for 18 h, the cells were treated with AP736 (0 to 30 *μ*M) for 30 min and then further incubated with LPS (1 *μ*g/mL) for 6 (PGE_2_) or 24 (NO) hours. The effects of AP736 on the production of NO and PGE_2_ were determined by analyzing the levels of these compounds using Griess reagents and EIA kits as previously described [18, 19].

### 2.4. Neutralizing Activity against SNP-Derived Radicals

The radical-scavenging activity of AP736 was determined by measuring its ability to neutralize the nitric oxide (NO^∙^) radicals released by spontaneous decomposition of SNP (10 *μ*M) for 30 min. The absorbances of the resultant chromophores were measured at 540 nm. Percent inhibition of NO generation was measured by comparing the absorbance values of negative controls (10 mM sodium nitroprusside and vehicle) to assay preparations.

### 2.5. Morphological Change Test

AP736-treated RAW264.7 cells, cultured in either the presence or absence of LPS, were incubated for 6 h. Images of the cultured cells at the designated time points were obtained using an inverted phase contrast microscope equipped with a video camera and captured using NIH image software as previously described [20]. A cell counter was used to determine the numbers of morphologically changed cells in each condition.

### 2.6. Determination of Phagocytotic Uptake

The phagocytic activity of RAW264.7 cells was determined essentially as described previously, but with minor modifications [21]. Briefly, RAW264.7 cells (5 × 10^4^) treated with AP736 were resuspended in 100 *μ*L PBS containing 1% human AB serum. Cells were then incubated with fluorescein isothiocyanate- (FITC-) dextran (1 mg/mL) at 37°C for 30 min. Incubations were stopped by the addition of 2 mL ice-cold PBS containing 1% human serum and 0.02% sodium azide. The cells were then washed three times with cold PBS-azide and analyzed on a FACScan flow cytometer as previously described [22].

### 2.7. Flow Cytometric Analysis

The level of FITC-dextran in RAW264.7 cells was determined by flow cytometric analysis [23, 24]. Briefly, RAW264.7 cells (2 × 10^6^ cells/mL) treated with AP736 in either the presence or absence of FITC-dextran (1 mg/mL) were washed with staining buffer [2% rabbit serum and 1% sodium azide in phosphate-buffered saline (PBS)] and then incubated with directly labelled antibodies for an additional 45 min on ice. After washing three times with staining buffer, stained cells were analysed on a FACScan flow cytometer (Becton-Dickinson).

### 2.8. Cell Viability Test

RAW264.7 cells (1 × 10^6^ cells/mL) were cultured for 18 h, after which time AP736 (0 to 30 *μ*M) was added to the cells for an additional 8 or 24 h of culture. The cytotoxic effect of AP736 was then evaluated by a conventional MTT assay as previously described [25, 26]. For the final 3 h of culture, 10 *μ*L MTT solution (10 mg/mL in phosphate-buffered saline, pH 7.4) was added to each well. Reactions were stopped by the addition of 15% sodium dodecyl sulfate (SDS) into each well, which solubilized the formazan products [27]. Absorbances at 570 nm (OD_570–630_) were measured using a SpectraMax 250 microplate reader (BioTek, Bad Friedrichshall, Germany).

### 2.9. mRNA Analyses Using Semiquantitative and Quantitative Reverse Transcriptase-Polymerase Chain Reactions

In order to determine the mRNA expression levels of various cytokines, total RNA was isolated from LPS-treated RAW264.7 cells using TRIzol reagent according to the manufacturer's instructions. Total RNA was stored at −70°C until use. Semiquantitative RT reactions were conducted as previously described [28]. Quantification of mRNA levels was performed by real-time RT-PCR using SYBR Premix Ex Taq according to the manufacturer's instructions (Takara, Shiga, Japan) and a real-time thermal cycler (Bio-Rad, Hercules, CA) as previously reported [29]. All primers used in this study were obtained from Bioneer (Daejeon, Korea) and are shown in Table 1.

### 2.10. Preparation of Cell Lysates, Preparation of Nuclear Fractions, and Immunoblot Analysis

RAW264.7 cells (5 × 10^6^ cells/mL) were washed three times in cold PBS supplemented with 1 mM sodium orthovanadate, resuspended in lysis buffer [20 mM Tris-HCl (pH 7.4), 2 mM EDTA, 2 mM ethyleneglycotetraacetic acid, 50 mM *β*-glycerophosphate, 1 mM sodium orthovanadate, 1 mM dithiothreitol, 1% Triton X-100, 10% glycerol, 10 *μ*g/mL aprotinin, 10 *μ*g/mL pepstatin, 1 mM benzamide, and 2 mM PMSF], and lysed by sonication with rotation for 30 min at 4°C. Lysates were clarified by centrifugation at 16,000 ×g for 10 min at 4°C and stored at −20°C until use.

Nuclear lysates were prepared following a three-step procedure [30]. After treatment as appropriate, cells were collected with a rubber policeman, washed with PBS, and lysed on ice for 4 min in 500 *μ*L lysis buffer containing 50 mM KCl, 0.5% Nonidet P-40, 25 mM HEPES (pH 7.8), 1 mM phenylmethylsulfonyl fluoride, 10 *μ*g/mL leupeptin, 20 *μ*g/mL aprotinin, and 100 *μ*M 1,4-dithiothreitol (DTT). Cell lysates were then centrifuged at 19,326 ×g for 1 min. For the second step, the nuclear fraction pellet was washed once in washing buffer (identical to the lysis buffer described above, except without Nonidet P-40). In the final step, the nuclei were treated with extraction buffer containing 500 mM KCl, 10% glycerol, and all other reagents included in the lysis buffer described above. The nuclei/extraction buffer mixture was frozen at −80°C, thawed on ice, and centrifuged at 19,326 ×g for 5 min. The resultant supernatant was collected as the nuclear extract. Soluble cell lysates were immunoblotted with the designated antibodies, and immunoreactive bands were visualized as previously reported [31].

### 2.11. Plasmid Transfection and Luciferase Reporter Gene Activity Assay

HEK293 cells (1 × 10^6^ cells/mL) were transfected with 1 *μ*g of plasmids driving the expression of *β*-galactosidase and either NF-*κ*B-Luc or AP-1-Luc in the presence or absence of an inducing molecule (MyD88 or TRIF). Transfections were performed using the PEI method in 12-well plates as previously outlined [32, 33]. Transfected cells were used at 48 h after transfection for all experiments. Cells were treated with AP736 for the final 8 h of each experiment. Luciferase assays were performed using the Luciferase Assay System (Promega, Madison, WI), as previously reported [34].

### 2.12. Statistical Analysis

All quantitative data are expressed as means ± standard deviations (SDs) as calculated from one of two independent experiments. Each experiment was done with 6 replicates. All other data shown are representative of three independent experiments. Statistical analyses were performed using analysis of variance/Scheffe's post hoc test and the Kruskal-Wallis/Mann-Whitney test. *P* values < 0.05 were considered statistically significant. All statistical tests were conducted using SPSS (SPSS Inc., Chicago, IL, USA).

## 3. Results and Discussion

In a previous study, AP736 was shown to downregulate the expression of tyrosinase by inhibiting the cAMP-PKA-CREB signalling pathway [17]. In addition, AP736 has also been reported to strongly suppress melanin production [16]. In the present study, we examined whether AP736 is able to modulate macrophage-mediated inflammatory responses in order to identify potential immunopharmacological roles for this compound.

**Figure 2:**
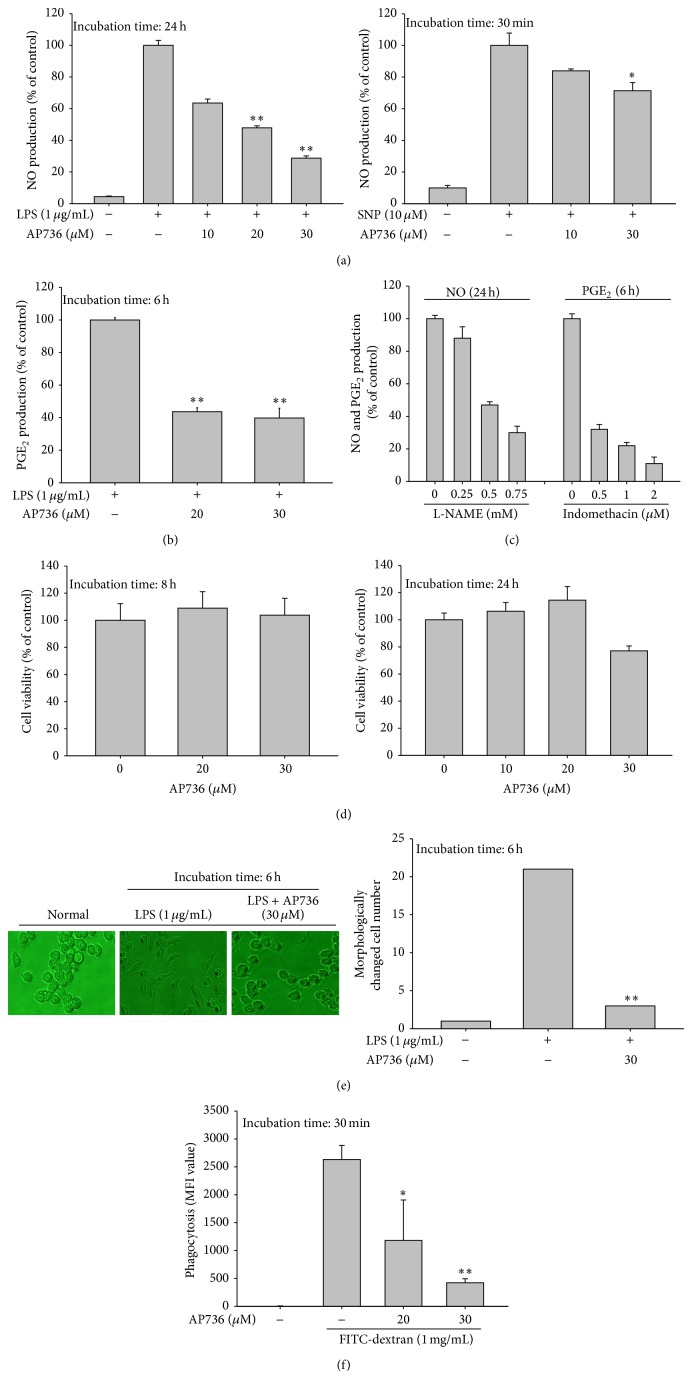
Effects of AP736 on macrophage-mediated inflammatory responses. ((a), (b), and (c)) The effects of AP736 or control compounds (L-NAME and indomethacin) on the levels of NO and PGE_2_ were determined in RAW264.7 cells (1 × 10^6^ cells/mL). Cells were treated with LPS (1 *μ*g/mL) in the presence or absence of AP736 for 24 or 6 h. After stimulation, culture supernatants were collected and the resultant concentrations of NO and PGE_2_ were determined using the Griess assay and EIAs (left panel). AP736 was incubated with SNP (10 *μ*M) for 30 min in microtubes. The levels of SNP-generated NO in microtubes were also determined by the Griess assay (right panel). (d) RAW264.7 cells (1 × 10^6^ cells/mL) were treated with AP736 for either 8 (left panel) or 24 (right panel) hours. Cell viability was evaluated using the MTT assay. (e) Images of the cells in culture at 6 h were obtained using an inverted phase contrast microscope equipped with a video camera and captured using NIH image software (left panel). Morphologically altered cells were enumerated with a cell counter (right panel). (f) RAW264.7 cells preincubated with AP736 were treated with FITC-dextran (1 mg/mL) for 30 min. The level of dextran uptake was determined by flow cytometric analysis. ^*^ *P* < 0.05 and ^**^ *P* < 0.01 compared with the normal or control groups.

To this end, we first tested the inhibitory activity of AP736 on NO and PGE_2_ production, two indicators of the extent of the inflammatory response [35], using LPS-treated RAW264.7 cells. As shown in Figures 2(a) and 2(b), AP736 effectively suppressed the production of both NO and PGE_2_. To determine whether AP736-mediated inhibition of NO release involves the direct scavenging of NO-derived radicals, we examined the ability of AP736 to inhibit SNP-derived NO release. As shown in the right panel of Figure 2(a), only marginal inhibition (25%) by AP736 was observed at 30 *μ*M, indicating that AP736-mediated suppression of NO production is due to not only direct radical scavenging by AP736, but also other pharmacological mechanisms. We also confirmed that two control compounds, L-NAME (used in the NO assay) and indomethacin (used in the PGE_2_ assay), exerted dose-dependent inhibition of NO release and PGE_2_ secretion, respectively (Figure 2(c)), consistent with previous results [36]. Importantly, AP736 did not show any cytoxicity to RAW264.7 cells at concentrations up to 30 *μ*M at 8 and 24 h (Figure 2(d) left and right panels). Cumulatively, these results clearly indicate that (1) our assays provide an accurate measure of the inflammatory response and (2) the inhibitory activity of AP736 is not simply due to nonspecific pharmacological activities.

Since we previously found that macrophage-mediated inflammatory responses are positively regulated by morphological changes mediated by the actin cytoskeleton [37, 38], we next examined whether LPS-induced morphological alterations are also affected by AP736. As shown in Figure 2(e), AP736 clearly suppressed LPS-induced structural alterations of macrophages by up to 90%, implying that the inhibitory activity of AP736 is also linked to the suppression of morphological changes in macrophages, as is the case for actin cytoskeleton-disrupting agents such as cytochalasin B [38]. In agreement with this hypothesis, AP736 also markedly suppressed the phagocytic uptake of RAW264.7 cells (Figure 2(f)). Phagocytic uptake is an extremely important part of the innate immune response and also in other inflammatory events. Since phagocytic uptake is also entirely dependent on actin cytoskeleton rearrangement [37], our results strongly imply that AP736 could suppress inflammatory responses by blocking actin cytoskeleton-dependent inflammatory events. The exact mechanism of how AP736 modulates actin cytoskeleton will be further explored in terms of actin and its regulatory proteins such as Rho A, Rac, and CDC42.

**Figure 3:**
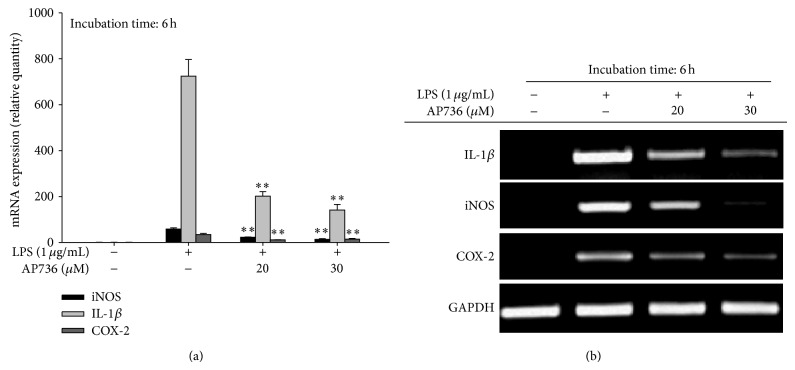
Effects of AP736 on the expression levels of inflammatory genes in LPS-treated RAW264.7 cells. (a) RAW264.7 cells (5 × 10^6^ cells/mL) were incubated with LPS (1 *μ*g/mL) in either the presence or absence of AP736 for 6 h. The mRNA levels of iNOS, COX-2, and IL-1*β* were then determined using real-time PCR. (b) RAW264.7 cells (5 × 10^6^ cells/mL) were incubated with LPS (1 *μ*g/mL) in either the presence or absence of AP736 for 6 h. The mRNA levels of iNOS, COX-2, and IL-1*β* were then determined using semiquantitative PCR. ^*^ *P* < 0.05 and ^**^ *P* < 0.01 compared with the control group.

Indeed, actin cytoskeleton rearrangement has been reported to be closely linked to the activation of NF-*κ*B. In fact, two IKK inhibitors, BAY 11-7082 and ginsenoside Rp1, have been shown to suppress actin cytoskeleton-dependent phagocytic uptake and cell-cell adhesion [39, 40], suggesting that inflammatory responses that rely on the actin cytoskeleton may be regulated by transcription factor (e.g., NF-*κ*B) activity. To determine whether AP736-mediated anti-inflammatory responses are mediated by the activation of transcription factors, we determined the expression levels of various inflammatory genes, such as IL-1*β*, iNOS, and COX-2. As expected, the expression of inflammatory genes was strongly downregulated in cells treated with either 20 or 30 *μ*M AP736, according to both real-time (Figure 3(a)) and semiquantitative (Figure 3(b)) RT-PCR analysis. Thus, these results strongly support the idea that AP736-mediated inhibition can be regulated at the transcriptional level. This type of regulation has also been reported for other known chemicals and inhibitors such as quercetin, lancemaside A, NSC95397, caffeic acid, the resveratrol derivative 2,4-dihydroxy-N-(4-hydroxyphenyl)benzamide, and lutein [39, 41–44].

**Figure 4:**
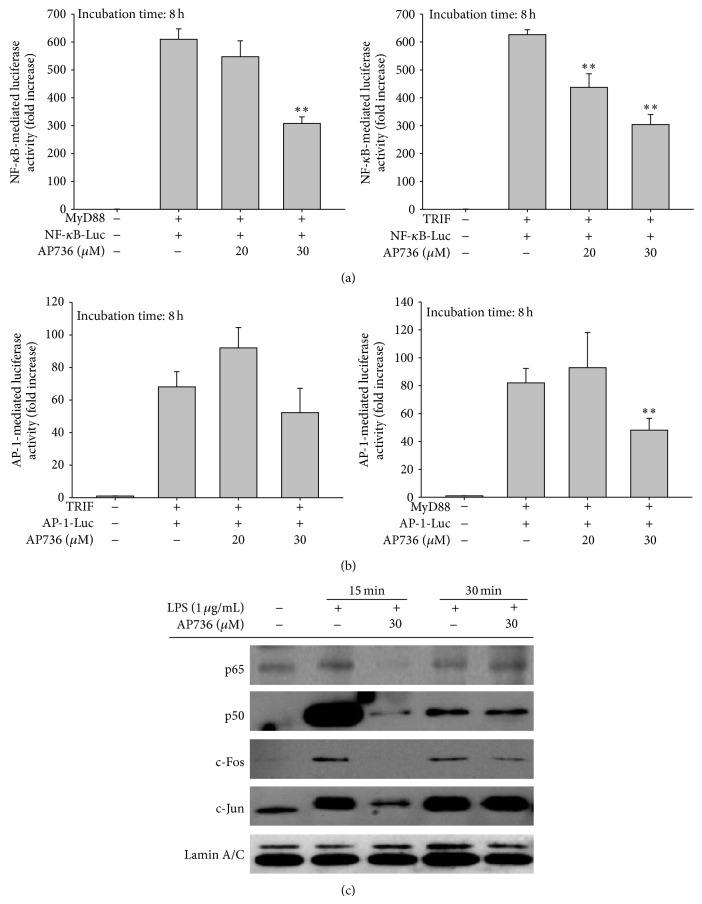
Effects of AP736 on the transactivation activity of transcription factors and their nuclear translocation. ((a) and (b)) HEK293 cells cotransfected with NF-*κ*B-Luc and AP-1-Luc constructs, as well as a *β*-gal construct as a transfection control, were treated with AP736 upon cotransfection with MyD88 or TRIF (1 *μ*g/mL each) for 8 h. The resultant luciferase activities were determined using luminometry as described in Section 2. (c) RAW264.7 cells (5 × 10^6^ cells/mL) were incubated with LPS (1 *μ*g/mL) in either the presence or absence of AP736 for the indicated times. After preparing nuclear fractions, the translocated levels of total or phosphorylated transcription factors (p65, p50, c-Fos, c-Jun, and lamin A/C) were identified using immunoblotting. ^*^ *P* < 0.05 and ^**^ *P* < 0.01 compared with the control group.

We next examined whether AP736 can also modulate the transcriptional activation of inflammatory transcription factors. For these experiments, we took advantage of the fact that transcription factor activity can be assessed with a luciferase reporter gene assay using cotransfection with TLR adaptor proteins (e.g., MyD88 and TRIF) [32, 45]. Interestingly, NF-*κ*B activation induced by TRIF was suppressed by 20 and 30 *μ*M of AP736, whereas MyD88-induced luciferase activity was only inhibited by 30 *μ*M of AP736 (Figure 4(a)). On the other hand, AP-1 activation upon MyD88 cotransfection, but not TRIF cotransfection, was inhibited by AP736 (30 *μ*M) (Figure 4(b)). These results imply that NF-*κ*B inhibitory activity of AP736 can be predominantly linked to the suppression of TRIF while inhibition of AP-1 activity could be marginally associated by blockade of MyD88 pathway. In fact, many signalling proteins have been reported to be activated by the overexpression of such adaptor molecules [32, 46]. The phosphorylation of NF-*κ*B and AP-1 subunits is known to be induced by cotransfection with MyD88 and TRIF [47, 48]. Therefore, TLR signalling pathways and the corresponding activation of transcription factors regulated by TLR adaptor molecules can be targeted by AP736. In agreement with our reporter gene assay, we also analysed nuclear extracts by immunoblotting and found that AP736 treatment strongly inhibited the nuclear translocation of p65, p50, and c-Jun at 15 min and c-Fos at 15 and 30 min (Figure 4(c)). The nuclear translocation of transcription factors from the cytosol is known to be mediated by the activation of these factors [49]. Thus, the observation that the nuclear levels of the NF-*κ*B and AP-1 subunits were decreased by AP736 indicates that these transcription factors could be targets of this compound.

**Figure 5:**
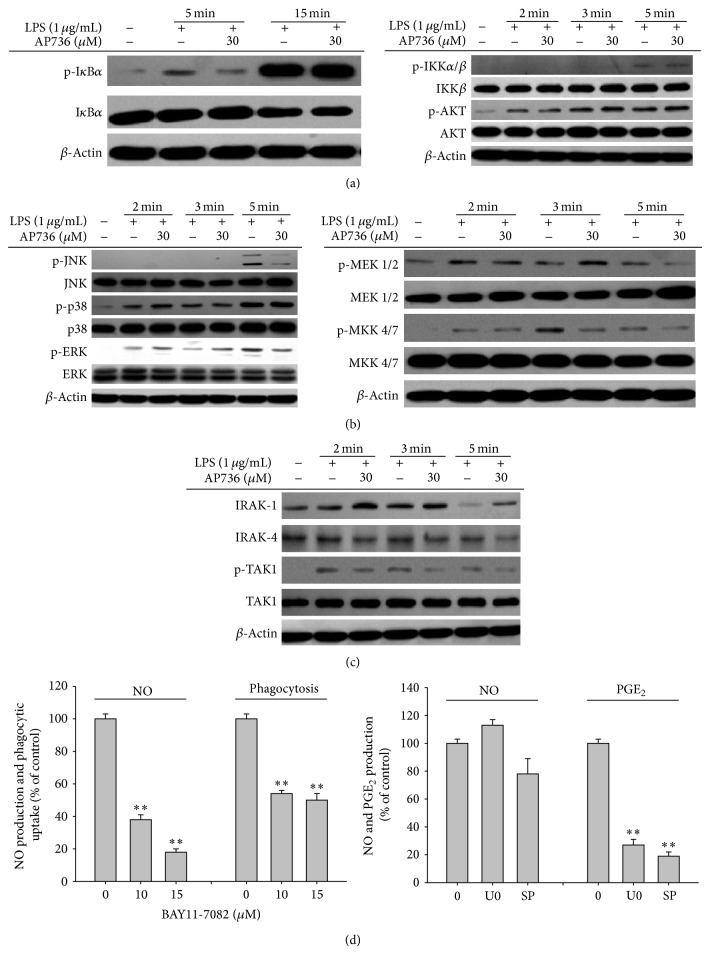
Effects of AP736 on signalling cascades upstream of NF-*κ*B and AP-1 activation. ((a), (b), and (c)) Phosphoprotein or total protein levels of I*κ*B*α*, IKK, AKT, p38, ERK, JNK, MEK1/2, MKK4/7, TAK1, IRAK1, IRAK4, and *β*-actin were determined by immunoblot analysis of cell lysates using phosphospecific or total protein antibodies. (d (left panel)) RAW264.7 cells (1 × 10^6^ cells/mL) were treated with FITC-dextran (1 mg/mL) or LPS (1 *μ*g/mL) in either the presence or absence of BAY 11-7082 (10 and 15 *μ*M). Cells were then incubated for 30 min (phagocytic uptake assay) or 24 h (NO assay). The levels of NO in culture supernatants were determined using the Griess assay. (d (right panel)) RAW264.7 cells (1 × 10^6^ cells/mL) were treated with LPS (1 *μ*g/mL) in the presence or absence of SP600125 (SP, 25 *μ*M) or U0126 (U0, 25 *μ*M). Cells were incubated for 6 h (PGE_2_ assay) or 24 h (NO assay). The levels of NO or PGE_2_ in culture supernatants were determined using the Griess assay and EIAs, respectively.

Numerous studies have indicated that nuclear translocation of inflammatory transcription factors is regulated by a variety of intracellular signalling networks. These signalling cascades include a series of activation events involving Syk, Src, PI3K, PDK1, AKT, IKK, and I*κ*B*α* for NF-*κ*B activation and IRAK1/4, TAK1, MKK, and MAPK (ERK, p38, and INK) for AP-1 activation [50–52]. Therefore, we also examined whether AP736 inhibits any of the intracellular signalling cascades that are known to affect both NF-*κ*B and AP-1 activation. To identify possible signalling events upstream of NF-*κ*B activation that are affected by AP736, the I*κ*B*α*/IKK pathway was first examined. As shown in Figure 5(a), the level of phosphorylated I*κ*B*α* was highly reduced after 5 min of treatment with AP736 (left panel), while other upstream phosphorylation events for IKK and AKT were not affected (right panel), indicating that IKK may be a target of AP736. We also evaluated the phosphorylation patterns of MAPK signalling proteins after treatment with AP736. As shown in Figure 5(b), the levels of phosphorylated JNK and ERK were decreased after 5 min of treatment with AP736. In agreement with these results, both the phosphorylation and degradation of signalling proteins upstream of MAPK (MEK1/2, MKK4/7, TAK1, and IRAK-1) were reduced by AP736, implying that AP736-mediated suppression of AP-1 may be mediated by the targeting of IRAK1. Taken together, these data clearly indicate that the activities of NF-*κ*B and AP-1 can be suppressed by AP736 via inhibition of upstream signalling pathways, including IKK/I*κ*B*α* and IRAK1/TAK1. To more precisely clarify the dual inhibitory actions of AP736, kinase assays with purified IKK and IRAK1 or luciferase assays employing the overexpression of IKK or IRAK1 that focus on subsequent downstream events will be performed in future studies.

**Figure 6:**
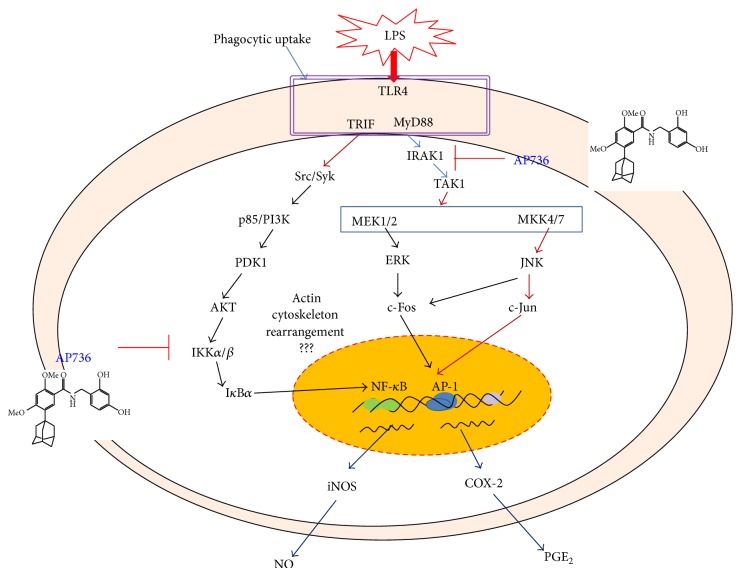
Putative pathway of AP736-mediated inhibition of anti-inflammatory responses.

In summary, our results indicate that AP736 can act as a potent anti-inflammatory drug. This compound remarkably suppressed multiple macrophage-mediated inflammatory responses, including NO/PGE_2_ production, inflammatory gene (iNOS, COX-2, and IL-1*β*) expression, phagocytic uptake, and morphological changes in activated macrophages. Analysis of potential target transcription factors revealed that NF-*κ*B and AP-1, in addition to their upstream signalling partners (IKK/I*κ*B*α* and IRAK1/TAK1, resp.), are dual immunopharmacological targets of AP736, as summarized in Figure 6. Based on these results, we conclude that AP736 can be either developed as an anti-inflammatory drug or further derivatized to improve its pharmacological activity, which would make it useful for the treatment of inflammation and/or pigmenting diseases of the skin.

## Figures and Tables

**Table 1:** Primer sequences used in RT-PCR and real-time PCR analysis.

Gene	Direction	Sequences
Real-time PCR		
IL-1*β*	F	5-GTTGACGGACCCAAAAAGAT-3
	R	5-CCTCATCCTGGAAGGTCCAC-3
iNOS	F	5-CCCTTCCGAAGTTTCTGGCAGCAGC-3
	R	5-GGCTGTCAGAGCCTCGTGGCTTTGG-3
COX-2	F	5-CACTACATCCTGACCCACTT-3
	R	5-ATGCTCCTGCTTGAGTATGT-3
GAPDH	F	5-CACTCACGGCAAATTCAACGGCAC-3
	R	5-GACTCCACGACATACTCAGCAC-3

RT-PCR		
IL-1*β*	F	5-CAGGATGAGGACATGAGCACC-3
	R	5-CTCTGCAGACTCAAACTCCAC-3
iNOS	F	5-CCCTTCCGAAGTTTCTGGCAGCAGC-3
	R	5-GGCTGTCAGAGCCTCGTGGCTTTGG-3
COX-2	F	5-CACTACATCCTGACCCACTT-3
	R	5-ATGCTCCTGCTTGAGTATGT-3
GAPDH	F	5-CAATGAATACGGCTACAGCA-3
	R	5-AGGGAGATGCTCAGTGTTGG-3

## References

[1] Zhuang Y., Lyga J. (2014). Inflammaging in skin and other tissues—the roles of complement system and macrophage. *Inflammation & Allergy-Drug Targets*.

[2] Willenborg S., Eming S. A. (2014). Macrophages—sensors and effectors coordinating skin damage and repair. *Journal der Deutschen Dermatologischen Gesellschaft*.

[3] Kurokawa I., Danby F. W., Ju Q., Wang X., Xiang L. F., Xia L., Chen W., Nagy I., Picardo M., Suh D. H., Ganceviciene R., Schagen S., Tsatsou F., Zouboulis C. C. (2009). New developments in our understanding of acne pathogenesis and treatment. *Experimental Dermatology*.

[4] Kim J. (2005). Review of the innate immune response in acne vulgaris: activation of toll-like receptor 2 in acne triggers inflammatory cytokine responses. *Dermatology*.

[5] Suzuki Y., Ra C. (2009). Analysis of the mechanism for the development of allergic skin inflammation and the application for its treatment: aspirin modulation of IgE-dependent mast cell activation: role of aspirin-induced exacerbation of immediate allergy. *Journal of Pharmacological Sciences*.

[6] Yoshizumi M., Nakamura T., Kato M., Ishioka T., Kozawa K., Wakamatsu K., Kimura H. (2008). Release of cytokines/chemokines and cell death in UVB-irradiated human keratinocytes, HaCaT. *Cell Biology International*.

[7] Harvima I. T., Nilsson G. (2011). Mast cells as regulators of skin inflammation and immunity. *Acta Dermato-Venereologica*.

[8] Toncic R. J., Lipozencic J., Martinac I., Greguric S. (2011). Immunology of allergic contact dermatitis. *Acta Dermatovenerologica Croatica*.

[9] Vestergaard C., Kirstejn N., Gesser B., Mortensen J. T., Matsushima K., Larsen C. G. (2001). IL-10 augments the IFN-*γ* and TNF-*α* induced TARC production in HaCaT cells: a possible mechanism in the inflammatory reaction of atopic dermatitis. *Journal of Dermatological Science*.

[10] Lee J.-W., Kim N. H., Kim J.-Y., Park J.-H., Shin S.-Y., Kwon Y.-S., Lee H. J., Kim S.-S., Chun W. (2013). Aromadendrin inhibits lipopolysaccharide-induced nuclear translocation of NF-*κ*B and phosphorylation of JNK in RAW 264.7 macrophage cells. *Biomolecules and Therapeutics*.

[11] Sekine Y., Yumioka T., Yamamoto T., Muromoto R., Imoto S., Sugiyma K., Oritani K., Shimoda K., Minoguchi M., Akira S., Yoshimura A., Matsuda T. (2006). Modulation of TLR4 signaling by a novel adaptor protein signal-transducing adaptor protein-2 in macrophages. *Journal of Immunology*.

[12] Takeda K., Akira S. (2001). Roles of Toll-like receptors in innate immune responses. *Genes to Cells*.

[13] Youn C. K., Park S. J., Lee M. Y., Cha M. J., Kim O. H., You H. J., Chang I. Y., Yoon S. P., Jeon Y. J. (2013). Silibinin inhibits LPS-induced macrophage activation by blocking p38 MAPK in RAW 264.7 cells. *Biomolecules and Therapeutics*.

[14] Makino E. T., Mehta R. C., Banga A., Jain P., Sigler M. L., Sonti S. (2013). Evaluation of a hydroquinone-free skin brightening product using in vitro inhibition of melanogenesis and clinical reduction of ultraviolet-induced hyperpigmentation. *Journal of Drugs in Dermatology*.

[15] Guerrero D. (2012). Dermocosmetic management of hyperpigmentations. *Annales de Dermatologie et de Vénéréologie*.

[16] Baek H. S., Hong Y. D., Lee C. S., Rho H. S., Shin S. S., Park Y.-H., Joo Y. H. (2012). Adamantyl N-benzylbenzamide: new series of depigmentation agents with tyrosinase inhibitory activity. *Bioorganic and Medicinal Chemistry Letters*.

[17] Lee C. S., Jang W.-H., Park M., Jung K., Baek H. S., Joo Y. H., Park Y.-H., Lim K.-M. (2013). A novel adamantyl benzylbenzamide derivative, AP736, suppresses melanogenesis through the inhibition of cAMP-PKA-CREB-activated microphthalmia-associated transcription factor and tyrosinase expression. *Experimental Dermatology*.

[18] Cho J. Y., Baik K. U., Jung J. H., Park M. H. (2000). In vitro anti-inflammatory effects of cynaropicrin, a sesquiterpene lactone, from *Saussurea lappa*. *European Journal of Pharmacology*.

[19] Kim D. H., Chung J. H., Yoon J. S., Ha Y. M., Bae S., Lee E. K., Jung K. J., Kim M. S., Kim Y. J., Kim M. K., Chung H. Y. (2013). Ginsenoside Rd inhibits the expressions of iNOS and COX-2 by suppressing NF-*κ*B in LPS-stimulated RAW264.7 cells and mouse liver. *Journal of Ginseng Research*.

[20] Kim M.-Y., Cho J. Y. (2013). 20S-dihydroprotopanaxatriol modulates functional activation of monocytes and macrophages. *Journal of Ginseng Research*.

[21] Duperrier K., Eljaafari A., Dezutter-Dambuyant C., Bardin C., Jacquet C., Yoneda K., Schmitt D., Gebuhrer L., Rigal D. (2000). Distinct subsets of dendritic cells resembling dermal DCs can be generated in vitro from monocytes, in the presence of different serum supplements. *Journal of Immunological Methods*.

[22] Lee Y. G., Lee W. M., Kim J. Y. (2008). Src kinase-targeted anti-inflammatory activity of davallialactone from *Inonotus xeranticus* in lipopolysaccharide-activated RAW264.7 cells. *British Journal of Pharmacology*.

[23] Cho J. Y., Fox D. A., Horejsi V., Sagawa K., Skubitz K. M., Katz D. R., Chain B. (2001). The functional interactions between CD98, *β*1-integrins, and CD147 in the induction of U937 homotypic aggregation. *Blood*.

[24] Lee Y. G., Lee J., Cho J. Y. (2010). Cell-permeable ceramides act as novel regulators of U937 cell-cell adhesion mediated by CD29, CD98, and CD147. *Immunobiology*.

[25] Pauwels R., Balzarini J., Baba M., Snoeck R., Schols D., Herdewijn P., Desmyter J., De Clercq E. (1988). Rapid and automated tetrazolium-based colorimetric assay for the detection of anti-HIV compounds. *Journal of Virological Methods*.

[26] Roh Y. S., Kim H. B., Kang C.-W., Kim B. S., Nah S.-Y., Kim J.-H. (2010). Neuroprotective effects of ginsenoside Rg_3_ against 24-OH-cholesterol-induced cytotoxicity in cortical neurons. *Journal of Ginseng Research*.

[27] Kim J. R., Oh D.-R., Cha M. H., Pyo B. S., Rhee J. H., Choy H. E., Oh W. K., Kim Y. R. (2008). Protective effect of polygoni cuspidati radix and emodin on Vibrio vulnificus cytotoxicity and infection. *Journal of Microbiology*.

[28] Lee Y. G., Chain B. M., Cho J. Y. (2009). Distinct role of spleen tyrosine kinase in the early phosphorylation of inhibitor of *κ*B*α* via activation of the phosphoinositide-3-kinase and Akt pathways. *International Journal of Biochemistry and Cell Biology*.

[29] Kang G.-J., Han S.-C., Ock J.-W., Kang H.-K., Yoo E.-S. (2013). Anti-inflammatory effect of quercetagetin, an active component of immature Citrus unshiu, in HaCaT human keratinocytes. *Biomolecules and Therapeutics*.

[30] Byeon S. E., Lee Y. G., Kim B. H., Shen T., Lee S. Y., Park H. J., Park S.-C., Rhee M. H., Cho J. Y. (2008). Surfactin blocks NO production in lipopolysaccharide-activated macrophages by inhibiting NF-*κ*B activation. *Journal of Microbiology and Biotechnology*.

[31] Lee J. Y., Lee Y. G., Yang K.-J. (2010). Akt Cys-310-targeted inhibition by hydroxylated benzene derivatives is tightly linked to their immunosuppressive effects. *The Journal of Biological Chemistry*.

[32] Shen T., Lee J., Park M. H. (2011). Ginsenoside Rp1, a ginsenoside derivative, blocks promoter activation of iNOS and COX-2 genes by suppression of an IKK*β*-mediated NF-*κ*B pathway in HEK293 cells. *Journal of Ginseng Research*.

[33] Song S. B., Tung N. H., Quang T. H., Ngan N. T., Kim K. E., Kim Y. H. (2012). Inhibition of TNF-*α*-mediated NF-*κ*B transcriptional activity in HepG2 cells by dammarane-type saponins from Panax ginseng leaves. *Journal of Ginseng Research*.

[34] Jung K. K., Lee H. S., Cho J. Y., Shin W. C., Rhee M. H., Kim T. G., Kang J. H., Kim S. H., Hong S., Kang S. Y. (2006). Inhibitory effect of curcumin on nitric oxide production from lipopolysaccharide-activated primary microglia. *Life Sciences*.

[35] Murakami A., Ohigashi H. (2007). Targeting NOX, INOS and COX-2 in inflammatory cells: chemoprevention using food phytochemicals. *International Journal of Cancer*.

[36] Jeong D., Yi Y.-S., Sung G.-H., Yang W. S., Park J. G., Yoon K., Yoon D. H., Song C., Lee Y., Rhee M. H., Kim T. W., Kim J.-H., Cho J. Y. (2014). Anti-inflammatory activities and mechanisms of Artemisia asiatica ethanol extract. *Journal of Ethnopharmacology*.

[37] Lee J. J., Kim D. H., Kim D. G. (2013). Toll-like receptor 4-linked janus kinase 2 signaling contributes to internalization of *Brucella abortus* by macrophages. *Infection and Immunity*.

[38] Kim J. Y., Lee Y. G., Kim M.-Y., Byeon S. E., Rhee M. H., Park J., Katz D. R., Chain B. M., Cho J. Y. (2010). Src-mediated regulation of inflammatory responses by actin polymerization. *Biochemical Pharmacology*.

[39] Kim E., Yang W. S., Kim J. H., Park J. G., Kim H. G., Ko J., Hong Y. D., Rho H. S., Shin S. S., Sung G.-H., Cho J. Y. (2014). Lancemaside A from *Codonopsis lanceolata* modulates the inflammatory responses mediated by monocytes and macrophages. *Mediators of Inflammation*.

[40] Byung H. K., Jae Y. C. (2009). Regulatory role of ginsenoside Rp1, a novel ginsenoside derivative, on CD29-mediated cell adhesion. *Planta Medica*.

[41] Endale M., Park S.-C., Kim S., Kim S.-H., Yang Y., Cho J. Y., Rhee M. H. (2013). Quercetin disrupts tyrosine-phosphorylated phosphatidylinositol 3-kinase and myeloid differentiation factor-88 association, and inhibits MAPK/AP-1 and IKK/NF-κB-induced inflammatory mediators production in RAW 264.7 cells. *Immunobiology*.

[42] Yang W. S., Jeong D., Yi Y.-S. (2013). IRAK1/4-targeted anti-inflammatory action of caffeic acid. *Mediators of Inflammation*.

[43] Kim M. H., Son Y.-J., Lee S. Y., Yang W. S., Yi Y.-S., Yoon D. H., Yang Y., Kim S. H., Lee D., Rhee M. H., Kang H., Kim T. W., Sung G.-H., Cho J. Y. (2013). JAK2-targeted anti-inflammatory effect of a resveratrol derivative 2,4-dihydroxy-N-(4-hydroxyphenyl)benzamide. *Biochemical Pharmacology*.

[44] Oh J., Kim J. H., Park J. G., Yi Y.-S., Park K. W., Rho H. S., Lee M.-S., Yoo J. W., Kang S.-H., Hong Y. D., Shin S. S., Cho J. Y. (2013). Radical scavenging activity-based and AP-1-targeted anti-inflammatory effects of lutein in macrophage-like and skin keratinocytic cells. *Mediators of Inflammation*.

[45] Kim M. H., Yoo D. S., Lee S. Y., Byeon S. E., Lee Y. G., Min T., Rho H. S., Rhee M. H., Lee J., Cho J. Y. (2011). The TRIF/TBK1/IRF-3 activation pathway is the primary inhibitory target of resveratrol, contributing to its broad-spectrum anti-inflammatory effects. *Pharmazie*.

[46] Chen R.-J., Yuan H.-H., Zhang T.-Y., Wang Z.-Z., Hu A.-K., Wu L.-L., Yang Z.-P., Mao Y.-J., Ji D.-J., Zhu X.-R. (2014). Heme oxygenase-2 suppress TNF-α and IL6 expression via TLR4/MyD88-dependent signaling pathway in mouse cerebral vascular endothelial cells. *Molecular Neurobiology*.

[47] Teng G.-G., Wang W.-H., Dai Y., Wang S.-J., Chu Y.-X., Li J. (2013). Let-7b is involved in the inflammation and immune responses associated with *Helicobacter pylori* infection by targeting Toll-like receptor 4. *PLoS ONE*.

[48] Yang Y., Yang W. S., Yu T., Yi Y. S., Park J. G., Jeong D., Kim J. H., Oh J. S., Yoon K., Kim J. H., Cho J. Y. (2014). Novel anti-inflammatory function of NSC95397 by the suppression of multiple kinases. *Biochemical Pharmacology*.

[49] Zanella F., dos Santos N. R., Link W. (2013). Moving to the core: spatiotemporal analysis of forkhead Box O (FOXO) and nuclear factor-*κ*B (NF-*κ*B) nuclear translocation. *Traffic*.

[50] Byeon S. E., Yi Y.-S., Oh J., Yoo B. C., Hong S., Cho J. Y. (2012). The role of Src kinase in macrophage-mediated inflammatory responses. *Mediators of Inflammation*.

[51] Yu T., Yi Y.-S., Yang Y., Oh J., Jeong D., Cho J. Y. (2012). The pivotal role of TBK1 in inflammatory responses mediated by macrophages. *Mediators of Inflammation*.

[52] Lee Y. G., Lee J., Byeon S. E., Yoo D. S., Kim M. H., Lee S. Y., Cho J. Y. (2011). Functional role of Akt in macrophage-mediated innate immunity. *Frontiers in Bioscience*.

